# Patient-derived tumor models: a more suitable tool for pre-clinical studies in colorectal cancer

**DOI:** 10.1186/s13046-021-01970-2

**Published:** 2021-06-01

**Authors:** Giulia Rizzo, Andrea Bertotti, Simonetta Maria Leto, Stefania Vetrano

**Affiliations:** 1 Department of Biomedical Sciences, Humanitas University, Via Rita Levi Montalcini, Pieve Emanuele, 20090 Milan, Italy; 2 Laboratory of Translational Cancer Medicine, Candiolo Cancer Institute – FPO IRCCs, Candiolo, 10060 Torino, Italy; 3 Department of Oncology, University of Torino School of Medicine, Candiolo, 10060 Torino, Italy; 4 IBD Center, Department of Gastroenterology, Humanitas Clinical and Research Center-IRCCS, Rozzano, Milan, Italy

**Keywords:** Colorectal cancer, Tumor heterogeneity, Tumor microenvironment, Pre-clinical models, Patient-derived xenograft, Patient-derived organoids, Drug screening platform, Personalized medicine

## Abstract

Colorectal cancer (CRC), despite the advances in screening and surveillance, remains the second most common cause of cancer death worldwide. The biological inadequacy of pre-clinical models to fully recapitulate the multifactorial etiology and the complexity of tumor microenvironment and human CRC’s genetic heterogeneity has limited cancer treatment development. This has led to the development of Patient-derived models able to phenocopy as much as possible the original inter- and intra-tumor heterogeneity of CRC, reflecting the tumor microenvironment’s cellular interactions. Implantation of patient tissue into immunodeficient mice hosts and the culture of tumor organoids have allowed advances in cancer biology and metastasis. This review highlights the advantages and limits of Patient-derived models as innovative and valuable pre-clinical tools to study progression and metastasis of CRC, develop novel therapeutic strategies by creating a drug screening platform, and predict the efficacy of clinical response to therapy.

## Introduction

Colorectal cancer (CRC) remains the second most common cause of cancer death, estimating globally 1.8 million new cases and 900,000 deaths annually in 2018 [1, 2]. Despite advances in screening and surveillance, the number of individuals newly diagnosed has been expected to rise further. The American Cancer Society has announced 147,950 new cases in 2020 in the US population, not only among the subjected aged 50 years and above but also younger adults [3, 4], pointing out urgency to respond to this upcoming CRC incidence, especially in developing countries.

**Fig. 1:**
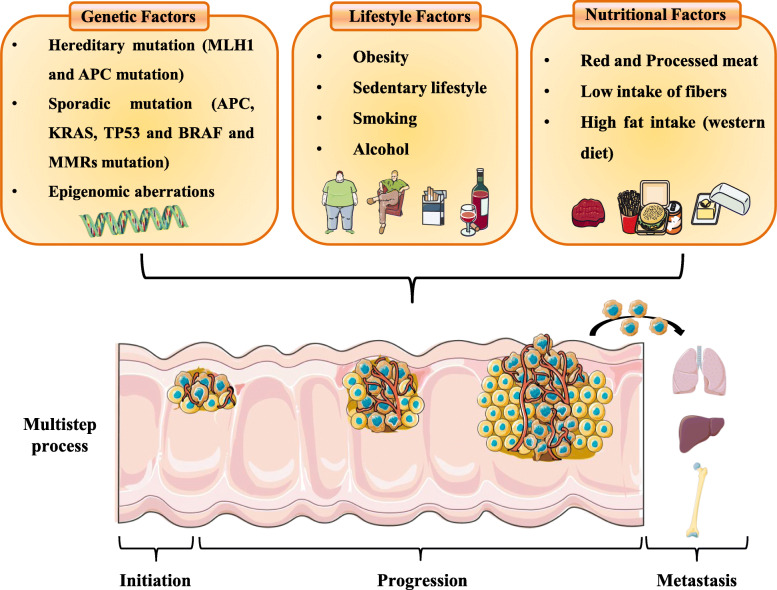
Genetic and environmental risk factors induce the CRC multistep process. The combination of genetic and environmental risk factors induces the CRC multistep process that determines the onset, progression, and metastasis of CRC. Approximately 30–40% of CRC have hereditary components (*MLH1* and *APC* mutations: hereditary non-polyposis CRC and familial adenomatous polyposis, respectively), while the 60% of CRC arise sporadically as a result of a combination of somatic genetic mutation in CRC driver genes such as *APC*, *KRAS*, *TP53* and *BRAF* or in DNA mismatch repair genes and epigenetic aberrations. Moreover, the environmental factors relating to lifestyle, particularly obesity, low physical activity, heavy consumption of alcohol and smoking, and nutritional factors, characterized by high consumption of red and processed meat and fatty foods, contribute overall to increase CRC risk

Only 2 to 5% of CRCs are hereditary cancer syndromes (such as Lynch syndrome and familial adenomatous polyposis (FAP) [2, 5], whereas the majority of CRCs (60–65%) arise sporadically as a result of a combination of somatic genetic and epigenetic aberrations, gut dysbiosis, chronic intestinal inflammation, lifestyle and environmental risk factors (e.g., cigarette smoking, excess body weight, alcohol intake, physical inactivity, and diet) [2, 6, 7] The combination of genetic and environmental risk factors contribute to the CRC multistep process characterized by the onset, the progression and the metastasis of CRC (Fig. 1). Nevertheless, the molecular mechanisms underlying these complicated interactions driving CRC development are not entirely clear. The multifactorial etiology reflects a heterogeneous disease characterized by different molecular features and responses to therapy, making the research for new therapeutic strategies an ongoing challenge. The considerable intra- and inter- tumoral phenotypic heterogeneity, due to an accumulation of multiple genetic alterations and genetic background of patients conditionate tumor microenvironment and tumor phenotype [2, 8]. The mutual interaction between the cancer cells, which trigger significant molecular and cellular changes within the host tissue, and resident host cells, including immune cells and stromal cells, supports tumor growth, progression, and survival [9–11]. Approximately 85% of CRCs are Microsatellite Stable (MSS) with chromosomal instability, while 15% of cases present Microsatellite Instability (MSI) with the defective function of mismatch repair DNA system [12–14]. In association with genetic mutations in genes like *RAS*, *RAF*, and *BRAF*, MSS and MSI features contribute to defining CRC subtypes, which further impact disease and response to therapy [4, 13, 15–21]. Despite the advances of therapeutic strategies, most new treatments fail upon reaching phase III clinical trials due to the lack of efficacy [22, 23]. The primary factor that plays a critical role in clinical trial failure is the biological inadequacy of pre-clinical models where drugs are developed or tested, capable of predicting humans’ therapeutic efficacy [22–25]. To date, the most critical question remains to identify the best predictor of therapy success in these patients for personalized treatment [4, 26, 27].

This review summarizes the strengths of the patient-derived models as the most suitable pre-clinical models able to phenocopy the original inter- and intra-tumor heterogeneity of CRC, to study the development and the progression of CRC, and to develop and test new therapeutic strategies. It also discusses these models’ criticisms and weaknesses to recapitulate the complexity of the tumor microenvironment and recent studies that have evoked promises to overcome some of these limitations.

## CRC pre-clinical models

Human cancer cell lines are in vitro model systems commonly used in basic cancer research and drug discovery. More than 100 cell lines have been recorded as CRC cell lines from different worldwide cell line banks. Nevertheless, few of these are entirely able to recapitulate the mutational and transcriptional heterogeneity of primary tumors [28] and are implied for studying functional biological mechanisms of CRC and pharmacogenomics. Based on the genes they express, CRC cell lines have been classified into six unique subtypes with diverse clinical features. Therefore, the choice of an appropriate subtype is critical for the scope of the studies. Moreover, despite these similitudes with the primary CRC, the manipulation in vitro for years following several passages leads to a divergence of cell lines from the original tumor. Furthermore, their artificial culture conditions that result in genetic, epigenetic, and transcriptomic changes during serial passaging with enrichment for specific sub-clones, the lack of the complex and continuous interaction with human immune and stromal compartment of the tumor microenvironment, profoundly impact the behavior of the cells leading to lack of experimental reproducibility and clinical relevance [29–35]. The in vivo experimental models of CRC have overcome the in vitro system’s limits and allowed us to improve our knowledge of cancer biology and identify novel therapeutic targets. In the early 1990s, the generation of genetically engineered mouse models (GEMMs) bearing nonsense mutations in the *Apc* gene that develop spontaneously multiple intestinal neoplasias similar to the FAP patients [36] has contributed enormously to the understanding of the molecular pathways involved in the early stages of tumor development. Nevertheless, the heterozygous mutation in the *Apc* gene (*Apc*^Min/+^) recapitulates the small intestine’s tumor lesions, but not those affecting the large colon resulting in an inappropriate model of sporadic colon-rectal cancer and for studying the metastatic processes. Transgenic mice with alternative *Apc* mutations and/or in combinations with other mutated suppressors or oncogenes such as *Ras*, *Cdx2*, *Tgfb*, *Pten*, *Smad3*, and *Braf* have been permitted to increase malignancy and tumor development also in the large colon and rectum. However, the drastic reduction of lifespan in mice bearing multiple mutations has limited these models’ use [37, 38]. *Apc*^*Min/+*^ mice treated with a carcinogenic agent such as azoxymethane (AOM) improve malignancy in the colon with the progression of adenocarcinoma lesions. Still, it increases the complexity of the CRC model and consequentially the molecular mechanisms involved. An alternative mouse model is a carcinogenic-induced model. Repeated injections of AOM combined with cyclic oral administration of dextran sulfate sodium (DSS), which is a time and dose-specific manner, is the most common CRC model used to recapitulate the aberrant crypt foci–adenoma-carcinoma sequence that occurs in human CRC [39, 40]. The low costs, the high reproducibility, and the high feasibility have widely diffused the use of the chemically AOM/DSS model for studying CRC initiation and progression. While GEMMs and carcinogenic-induced models represent a valuable tool in basic cancer research and drug discovery, they cannot reproduce the multiple aspects of a clinical trial. The lack of a varied diet, lifestyle, and an equal microbiome that mimics humans can enormously impact the disease’s onset and course. Besides, the short lifespan of the mouse that limits tumor development and the lack of inter-tumoral heterogeneity due to poor genetic heterogeneity in inbred mice compared to the outbred human population limits the results’ enthusiasm their potential clinical translations.

## Patient-derived models

The need for a pre-clinical model that phenocopies the original tumor inter- and intra-heterogeneity and preserves the factors influencing the growth and development of human cancers has led to the generation of Patient-Derived Xenograft (PDX) based on the direct implantation of patient tumor tissue into immunodeficient mouse hosts. The process of generating PDXs in mice from primary or metastatic tumors has been widely reported in the literature [41–43] since the first report of a successful xenograft in 1953 [44]. This approach allows preservation of the parental tumor architecture and the existing interactions of cancer cells with stromal and immune cells [45–49]. Multiple studies have confirmed the high concordance between PDX and the corresponding primary tumor in histopathological and molecular features over several passages maintaining genetic stability [45, 47, 50–60]. However, a low percentage of mutated variants may arise through the passages in PDX [55–58]. The application of the consensus molecular subtype (CMS) classification of CRC tumors has been pointed out in PDXs CMS groups in the same way as the matched primary tumors, without variations of CMS frequencies [57, 59, 61]. The positive immunoreactions for cytokeratin 20 and carcinoembryonic antigen, patterns exclusive of CRC [58, 62], and negative for cytokeratin 7 further support that PDX retains histopathological characteristics of the original malignancies. The possibility to generate cultures of tumor cells that can be derived from cancer patients with a high success rate and expanded for several passages recapitulating morphological and genetic features of the original tumor has led to the increasing development of patient-derived organoids (PDOs) in recent years. Like PDX, cancer organoids are three-dimensional cultures derived from stem cells that mimic a high degree of similarity to the tissue of origin, preserving the inter-patient heterogeneity of CRC and mirroring the genetic and phenotypic characteristics of tumor epithelium [63, 64]. A comparative analysis of PDX, PDO, and the corresponding tumors using different approaches [45, 47, 51–58, 62, 65, 66], including next-generation sequencing (NGS), has revealed a high fidelity in mutational status between the matched tumor and Patient-derived models, recapitulating most of CRC somatic mutation in several genes including *APC*, *p53*, *KRAS*, *NRAS*, *BRAF*, *PIK3CA*, *PTEN* [45, 62, 67]. Notably, Janakiraman and collogues comparing PDX and the subsequent PDO from pre-neoadjuvant therapy rectal cancer [54] has evidenced a substantial overlap in their mutational profiles (> 80% congruent), identifying *APC* and *TP53* mutations in 83 and 78% of tumors, respectively and loss of heterozygosity of *TP53* gene that leads to inactivation of the tumor suppressor genes, stabilizing *TP53* mutation and promoting oncogenic gain of function activity [68–70] in 100% of tumors. Importantly, both PDX and PDO replicated the clinical therapeutic response observed in the corresponding patient tumors to a neoadjuvant therapy consisting of the combination of 5-fluorouracil (5-FU), a fundamental component of chemotherapeutic agents and a standard therapy for advanced tumor and radiotherapy (RT). Adding cetuximab to 5FU/RT therapy, the authors observed increased PDX and PDO sensitivity with wild-type *KRAS* compared to the mutated *KRAS*. Another study carried out by Vlachogiannis et al., through NSG analysis, revealed an overlapping of 96% of the mutational spectrum between PDOs and their parental tumor [63].

**Fig. 2:**
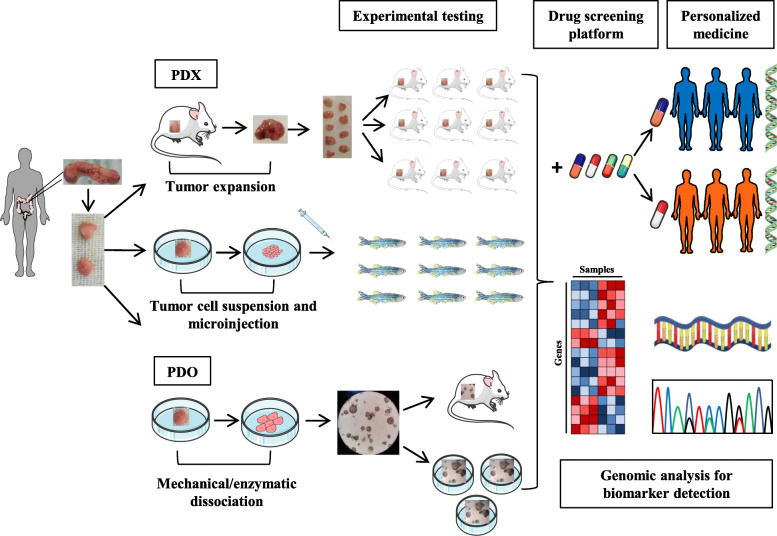
Schematic representation of Patient-derived model and their applications. Intestinal tumor sample derived from surgical resection is cut into small fragments and used for the generation of PDX and/or PDO. For the generation of the murine PDX model, the piece of the tumor is implanted subcutaneously into one or two flanks of an immunodeficient mouse. When the tumor expands, it is recovered, cut into smaller pieces, and implanted into new immunodeficient mice as recipients to generate experimental groups. To develop zPDX experimental groups, cell suspension derived from tumor patients is microinjected in zebrafish. For PDO model development, the piece of the tumor is dissociated mechanically and/or enzymatically, and the derived cells are embedded into Matrigel. PDOs can be implanted into immunodeficient mice and/or maintained in culture to generate experimental testing groups. Patient-derived models accurately reflect the patient's tumor of origin and can be exploited to generate a drug screening platform, to detect new tumor biomarkers by genomic analysis and to develop a new personalized treatment based on the patient’s genetic

Therefore, both PDX and PDO models reflecting the original tumor at both genomic and transcriptomic levels allow an increased number of possible applications for studying cancer biology, metastasis, and new treatment development (Fig.2). However it is noteworthy to consider the genomic drift that could occur over extensive passages (in vivo passages and in vitro culturing) [66, 71].

### Generation of PDX models

Although the first report of a successful xenograft was reported in 1953, only more recently, standards for the generation, quality assurance, and use of PDX models have been delineated [72].

Tumors, collected by surgery or biopsy procedures, are implanted directly after resection or after cryopreservation as pieces (25–30 mm^3^) or single-cell suspensions. Tissues properly cryopreserved maintain engraftment success and tumor growth [73, 74] without compromising cell viability. The tissue can be dipped in Matrigel, a solubilized tissue basement membrane matrix or mixed with human fibroblasts before implantation to enhance the engrafting outcome [73, 75]. Specimens can be implanted heterotopically, via subcutaneous implantation (s.c.) into the dorsal area of mice, or orthotopically, through direct transfer into the anatomical site of origin (colon cecal wall [76–78] or liver parenchyma [79]). In the field of CRC, s.c. is the most commonly used procedure for the generation of PDXs, since it is technically simple and facilitates tumor engraftment, monitoring, and resecting [80]. Advantages of orthoxenografts include the possibility to study local invasive growth of primary tumors, development of patient-like metastases [76, 81], tumor-host interactions in their anatomical context, and site-specific dependence on therapy. However, considerable microsurgical skills and small animal imaging modalities (e.g., tomography equipment) to visualize tumors are required [48], making this approach less easy than other pre-clinical animal models. The median overall engraftment rate for CRCs is 70% [82], and establishment typically takes 2–4 months [83]. These parameters may vary depending on sample type (e.g., engraftment rate is higher in surgical rather than biopsy specimens [50]), tumor subtype and stage [59], and recipient strain [84]. In a survey on 33 CRC PDXs, Prasetyanti et al. observed that epithelial subtypes (CMS2 and CMS3) displayed lower engraftment take rate than MSI-immune (CMS1) and mesenchymal (CMS4) tumors; however, further studies with larger datasets are required to validate these observations statistically [59]. Moreover, the engraftment success strongly depends on the tumor stage, with higher take rates from metastatic samples than primary tumors [59, 85].

Immunodeficient mice such as NOD/SCID (Nonobese diabetic/severe combined immunodeficiency) and NOD/SCID/IL2γ-receptor null (NSG) are the most suitable hosts for PDX generation owing to their lower immune rejection and higher engraftment rates [43, 84, 86, 87]. However, in addition to the defects of the innate and adaptive immune system, NOD strains express the human-like signal regulatory protein alpha (*Sirpa*) locus in macrophages and myeloid cells, leading to the interaction with its human ligand CD47 and generating a “don’t-eat-me” signal [88, 89].

### Translational applications of PDX models

PDXs are promising models for addressing clinically relevant objectives such as drug repurposing, prediction of clinical outcomes, identification of biomarkers of sensitivity and resistance to treatments, and understanding how tumor heterogeneity and clonal dynamics evolve during tumor progression and influence therapeutic responsiveness [48]. The high concordance of drug responses between patients and PDXs [45, 65, 90, 91] supports these models’ use as screening platforms to investigate new therapeutic options (Xeno trials). The generation of large mice cohorts derived from the same tumor sample allows to perform genotype-response correlations and overstep the number of testable hypotheses in humans [48]. This approach helps select patients subgroups likely to benefit from alternative therapies and prioritize new biomarkers development [43]. In a proof-of-concept study, the amplification of Erb-B2 Receptor Tyrosine Kinase 2 (*ERBB2*) gene, an oncogenic driver and a prognostic and predictive biomarker in CRC [92], was identified as a driver of cetuximab resistance in *KRAS/NRAS/BRAF/PI3KCA* wild-type metastatic CRC PDXs, and was found to predict response to Epidermal Growth Factor Receptor (*EGFR*) and Human Epidermal Growth Factor Receptor 2 (*HER2*) targeted therapies [45]. These findings were translated into successful clinical studies [93–95]. Similarly, by candidate-gene or comprehensive genomic analyses, other actionable targets were identified as cetuximab-resistance biomarkers, including MET Proto-Oncogene and Fibroblast Growth Factor Receptor 1 (*FGFR1*) amplification [46, 96], *ERBB2* and *MAP2K1* activating mutations [46, 97], insulin Like Growth Factor 2 (*IGF2*) overexpression [98], and the fusion of echinoderm microtubule-associated protein-like 4 (*EML4*) gene with the anaplastic lymphoma kinase (*ALK*) gene leading to the production of a protein (EML4-ALK) that promotes and maintains the malignant behavior of the cancer cells [99]. More empirically, Hinze and colleagues showed that CRC PDXs displayed significant tumor responses upon Glycogen Synthase Kinase 3 α (GSK3α) inhibition combined with the anti-leukemic enzyme asparaginase [100]. However, further studies are required before the translation of such results into viable therapeutic approaches. Recently, direct drug testing on PDXs has been complemented by functional genomics approaches. ShRNA libraries have been used to identify tumor vulnerabilities in melanoma and pancreatic cancer PDXs [101, 102]. CRISPR/Cas9 protocols have been exploited for direct in vivo functional genomics in proof-of-concept studies [103, 104]. When performing drug discovery and biomarker identification studies, PDXs have the potential to better recapitulate the inter-patient heterogeneity of cancer over traditional cell lines thanks to the possibility to conduct real population-scale studies. Therefore, large-scale PDX trial formats, such as the PDX Encyclopedia, are better suited to accurately predict clinical trial responses and capture therapeutic candidates [90]. Moreover, the statistical robustness of PDX data can be further reinforced by the emergence of international multi-institutional collaborations (e.g., the EurOPDX consortium) voted at tackling translational challenges [43, 48, 80]. PDX represents a promising tool to identify personalized therapy to treat CRC. Altunel et al., performed high-throughput drug screening composed of 119 FDA-approved oncology drug libraries and tested the response to standard chemotherapeutic agents in CRC PDX and the matched Patient-derived cell lines. The authors found a similar response to standard-of-care agents in the matched cell lines and PDX, allowing rapid analysis of sensitivity and resistance to standard-of-care agents. Moreover, from drug screening data, ponatinib, a multi-kinase inhibitor targeting several factors, including FGFR, platelet-derived growth factor receptor, vascular endothelial growth factor receptor, proto-oncogene tyrosine-protein kinases Src and Abl, was identified as a potentially effective therapeutic target for CRC for its action in inhibiting cell growth [105]. PDX model allows investigating the efficacy of the controversial anti-tumor agent. Indeed, in metastatic CRC (mCRC) metformin, accumulating in *KRAS*-mutated tumor cells inhibited tumor growth and cell viability. *KRAS* mutation induced the silencing of MATE1 (multidrug and toxic compound extrusion 1), a pump responsible for the efflux of metformin from the tumor cells, through the upregulation of DNMT1 [106]. Beyond identifying predictive biomarkers, PDXs may also facilitate understanding adaptive escape mechanisms sustaining residual disease at maximal drug response. Recent findings indicate that mCRC cells surviving EGFR-targeted therapy in PDXs display phenotypic reprogramming, characterized by reduced expression of EGFR ligands and high HER2/HER3 signaling. In Xeno trials, Pan-HER antibodies minimized residual disease and delayed tumor relapse after treatment suspension [107]. Interestingly, Kreso et al. demonstrated that chemotherapy treatment does not select for novel genetic clones in CRC PDXs; instead, actively proliferating cells were preferentially eradicated, while relatively dormant persisters became dominant [108]. These findings highlight the contribution of non-genetic processes sustaining cell heterogeneity and chemotherapeutic tolerance in CRCs [108]. Therefore, tumor responses should be evaluated by merely measuring tumor sizes and monitoring clonal selection and dynamics and functional imaging [109]. On the debated effect of anti-EGFR therapy on patients with the *KRAS*^G13D^ mutation, cetuximab therapeutic efficacy was evaluated on the *KRAS*^G13D^ CRC PDX model dissecting the potential resistance mechanism. After repeated treatment tumor acquired resistance to cetuximab, and significant changes were identified in JAK2, PRKAA1, FGFR2, and RALBP1 expression. In particular, *SWAP70*, related to tumor development, may be a probable gene involved in cetuximab resistance in* KRAS*^G13D^ CRC [110].

Of course, besides functional adaptation, tumor cells’ chances to survive therapy strongly depend on pre-existing intra-tumor heterogeneity [111]. Within this context, concerns have been raised about PDXs, based on the idea that artifactual selection processes can specifically affect the grafting process in mice [71]. However, although some clonal selection on initial engraftment may occur [71, 112, 113], the intra-tumor clonal architecture of the original tumors is preserved upon serial passaging in xenografts [108, 114, 115], supporting the notion that PDXs can be effectively exploited to investigate cancer clonal evolution [116]. Another application of PDXs stems from the possibility to simultaneously test anticancer drugs in patients and PDXs with similar genetic abnormalities (PDX co-clinical trials) to allow real-time integration of information [117].

Specifically, PDX models derived from cancer patients enrolled in clinical trials and treated with the same agents are called “avatars” [43, 118]. Although technically challenging and time-consuming [109], co-clinical avatar trials in CRCs are currently ongoing [119, 120] and already generated promising results [121, 122]. For example, in *BRAF* mutant CRCs, avatars showed drug responses that closely mirrored those in the corresponding patients [121] and allowed to investigate acquired resistance mechanisms [122]. PDX is a promising model for testing novel immunotherapy approaches such as Chimeric Antigen Receptor T (CAR-T)-cell, genetically engineered T cells that recognize cancer cells due to the expression of the antigen-specific receptor [123, 124]. Teng et al. tested on the HER2^+^ CRC PDX model the efficacy of HER2-specific CAR-T cells that infiltrate into the tumor, selectively eliminated the HER2^+^ cells causing the decrease in tumor size and the complete tumor elimination after 2 months from treatment [125]. Of course, the lack of an immune-competent environment hampers the utility of conventional PDXs. To overcome this limitation, approaches to generate “humanized mice” have been developed [48]. By selected immune components transplantation in mice, the efficacy of different immunotherapies can be explored [126]. Although implying several drawbacks [48, 127–129], humanization procedures have achieved promising results in CRC PDXs, in line with several clinical findings [130–132]. PDX model applications, outcome, and limits are summarized in Table 1. In recent years an emerging alternative to the murine PDX model is the zebrafish PDX (zPDX) to perform in vivo therapeutic screening and predict tumor response to therapy. In zPDX CRC cell lines and cell suspension derived from the patient, biopsies were microinjected, without in vitro passaging, into multiple zebrafish larvae, and the therapy response was evaluated in just 4 days (Fig. 2) [136–138]. Increasing shreds of evidences demonstrated that the zPDX were able to recapitulate several cancer features such as proliferation, metastatic potential and angiogenesis and to predict tumor responses to radiotherapy [136] and standard chemotherapy, such as FOLFOX (5-FU + oxaliplatin + folinic acid) and FOLFIRI (5-FU + irinotecan + folinic acid) [137]. zPDX allowed to distinguish radiosensitive from radioresistant CRC clones and discriminate the tumor cells with different chemotherapy sensitivities. In particular HCT116 cells were sensitive to radiation and were the only ones to respond to the FOLFOX, inducing apoptosis and reducing tumor size due to the *KRAS* mutation (*KRAS*^G13D^) that sensitized cells to the chemotherapy. Neoadjuvant therapy was tested in zPDX generated from rectum cancer biopsies showing apoptosis induction that correlates with the matched patient clinical response. Moreover, evaluating tumor response to cetuximab, Hke3 *KRAS*^WT^ and HCT116* KRAS*^G13D^ cells responded to cetuximab, comparable with the clinical responses in those, in which a fraction of patients with *KRAS*^G13D^, but not *KRAS*^G12V^ mutation responded to the treatment [137]. Overall, these shreds of evidence highlight the zPDX as a rapid and highly sensitive model to perform in vivo screening of the main current therapy for CRC.

**Table 1:** Applications of CRC patient-derived models in translational research

CRC Patient-derived model	Applications	Outcome	Advantages	Challeges	References
**PDX**					
Primary or metastatic tumor implanted s.c.	Investigation of primary and acquired mechanisms of resistance	Amplification of ERBB2 gene as a driver of cetuximab resistance in KRAS/NRAS/BRAF/PI3KCA WT-metastatic CRC [45]; MET proto-Oncogene and FGFR1 amplification, ERBB2 and MAP2K1 activating mutation, IGF2 overexpression and EML4-ALK fusion protein production have been identified as cetuximab-resistance biomarkers [94–98]	Easy tumor monitoring and resecting	Slow expansion	[45, 46, 88, 94–98, 103, 104, 112]
	Discovery of prognostic and predictive biomarkers		Preserved intra-tumor and inter-patient heterogeneity	Difficult genetic manipulation	
	Identification of new actionable targets	Pharmacologic GSK3α inhibition is sufficient to sensitize APC or β-catenin-mutant CRC to the anti-leukemic enzyme asparaginase displaying major tumor response. GSK3α inhibiting WNT-activating mutations, such as the RSPO3 fusion may predict asparaginase sensitivity [98]	Maintainance of original tumor architecture	Large collections and HTS difficult to realize	
	Understanding of adaptive non-genetic processes sustaining residual disease	A reduced expression of EGFR ligands and high HER2/HER3 signaling have been displayed in mCRC cells surviving EGFR-targeted therapy. Pan-HER antibodies reduce residual desease and delay tumor relapse [103]		Lack of several immune system components	
	Characterization of tumor heterogeneity and clonal evolution	Chemotherapy treatment eradicate actively proliferating cells while the resistant cells become dominant leading to a chemotherapeutic tolerance in CRC [104]		Progressive loss of human stroma cells and their replacement by murine counterparts	
	Study tumor-stroma interactions				
Primary or metastatic tumor implanted orthotopically	Investigation of mechanisms of invasion and metastasis	The tumorigenic potential of CRC stem cells (CCSCs) isolated from fresh human CRC have been evaluated through the CCSCs othotopically implantation into the wall submucosa of the ascending colon. The formation of spontaneous metastatic lesions was observed at local and distant sites (liver and mesenteric lymph nodes). Circulating CCSCs derived from CCSCs implanted in the colon can infiltrate and sustain distant metastasis [74]	Local invasive growth and tumor-host interactions in proper anatomical contex	Microsurgical skills	[74–77, 79]
	Study site-specific dependence on therapy		Spontaneous patient-like metastases development	Small animal imaging equipment required for tumor monitoring	
Co-clinical trials and avatars	Real-time adaptive therapeutic decisions during clinical trials	Avatars with BRAF mutation show drug responses that mirror those in the corresponding patients, allowing to investigate the acquisition of resistance mechanisms [117, 118]	Best-matched PDX models for individual patients	Time-consuming	[117, 118]
				Not all tumor stages engraft	
Humanized PDX models	Study how immune cells influence tumor progression	Anti-PD-1 therapy inhibits the tumor growth in MSI-H CRC, correlated with the increase of CD8 T cells and INF-γ-producing CD8+ tumor-infiltrating leukocytes, while fails in MSS-CRC, reflecting the patient's clinical outcome [123]	Mimicred human immune system in mice	Early onset of graft-versus-host disease	[123]
	Investigation of immunotherapies			Difficult and risky procedures during human stem cells collection in patients	
**PDO**					
Normal and tumor organoid cultures	Drug development	Identification of CRC patients not responding to irinotecan-based chemotherapy [133]; ERBB2-amplified, but not EGFR-amplified, PDO respond to lapatinib [63]; Combined inhibition of EGFR and KRAS^G12C^ is effective against colorectal with KRAS^G12C^ mutations [134]; WNT inhibition can improve the outcome of the 5-FU-based therapy [135]	Ease of genetic manipulation, *in vitro* functional studies and HTS	Lack of blood vessels, stroma and immune cells	[63, 132–152]
	Personalized medicine	Identification of specific drug sensitivities or resistances for each patient. CRC with KRAS and TP53 mutations is sentitive to trametinib alone or in combination with several targeted agents (celecoxib). Afatinib and the other EGFR inhibitors are effective against CRC with mutations in APC mutations and more effective in combination with HDAC inhibitors [85]	Fast expansion		
	Modelling of cancer initiation and progression	Human colon organoids edited through CRISPR/Cas9 to induced mutation in tumor suppressor genes and oncogenes are tumorigenic *in vivo* but spontaneously develop metastasis only when implanted orthotopically into the naive microenvironment [148–151]	Healthy control organoids availability		
	Study of single-cell tumor heterogeneity and clonal dynamics	In CRC clonal organoids have been identified distinct clonal organoids derived from the same tumor region with different drug responses [147]	Retained intra-tumor heterogeneity		
			Possible transplantation in mice to substitute PDXs for *in vivo* studies		
Air-liquid interface and co-culture approaches	Interrogation of tumor cells interactions with stroma and immune system	Air-liquid interface (ALI) recapitulates the interaction between tumor cells (PDOs) and tumor microenvironment components (stromal and native immune cells) and functionally models the immune checkpoint blockade with anti-PD-1 and/or PD-L1 that activates the tumor cytotoxicity mediated by CD8^+^TIL [153]	Preservation of endogenous immune stroma	Lack of blood vessels	[153–157]
	Study of tumorigenesis	Exploiting the co-culture of mouse intestinal organoids and fibroblasts, a rare fibroblast subpopulation that regulates tumor-initiating stem cells have been observed [101]			
	Investigation of immunotherapies	Exploiting the co-culture of PDO derived from chemotherapy resistant mCRC and CD8 T cells, the efficacy of CEA-TCB immunotherapy have been evaluated. Low expression of CEA correlate with resistance to immunotherapy [137]			

### Generation of PDO models

While PDXs trials may be highly informative and expensive, timely long, and technically cumbersome, in vitro cultures of patient-derived cells have the potential to be more easily expanded and managed for genetic manipulations and high-throughput screenings (HTS) [158]. Moreover, the possibility of generating patient-derived cultures from both tumors and matched healthy tissues enables direct comparisons during molecular and functional studies and increases the number of possible applications.

However, this theoretical potential has been historically constrained by the low efficiency of primary cell line generation from human tumors [139]. Until recently, this has been true when protocols for the establishing PDOs have been optimized [133, 140].

In general, organoids derivation requires three major steps: tissue fragmentation (either mechanical or enzymatic), cells embedment into a 3D extracellular matrix substitute (Matrigel® or Basement Membrane Extract), and culture in serum-free medium supplemented with different stem cell niche factors [141, 142]. This approach allows self-organizing three-dimensional structures that resemble many structural and functional aspects of the original tissue [134, 140]. While healthy tissue intestinal organoids typically display budding elements [140], tumor-derived organoids range from thin-walled cysts to compact structures without a lumen [134, 135]. Supplements for long-term expansion of normal intestinal organoids include: Wnt-3a and R-spondin (Wnt activators important to maintain stem cell population), EGF (RTK ligand that promote cell proliferation), Noggin (BMP inhibitor that support stem cell expansion), gastrin (hormone), A83–01 (ALK inhibitor) and SB202190 (p38 inhibitor involved in apoptosis, proliferation and differentiation) [134, 140, 142, 143]. The substitution of the p38 inhibitor with the combination of IGF-2 and FGF-2 has improved culture efficiency and preserved cellular diversity [144].

Primary and metastatic CRC organoids can be established directly from patient specimens (biopsy or surgical resection) or PDX explants [99, 134, 135, 145, 159, 160]. Since tumor organoids’ niche factor dependency gradually decreases during tumor progression, less stringent culture conditions are required for CRC PDOs. Indeed, while EGF is still essential for the growth of the majority of metastatic CRC organoids, other factors are dispensable (i.e., Wnt/R-spondin and Noggin) or even detrimental (e.g., p38i) [159].

Reported CRC PDOs establishment rates range between 60 to 90% [63, 99, 135, 145, 146, 160] and correlate with tumor cellularity in the original sample [63, 135]. Moreover, intestinal organoids can be cultured for a long time and are amenable to cryopreservation [134].

### Translational applications of PDO models

Compared with conventional cell lines, PDO cultures offer the same experimental versatility with the advantages of retaining patient tumor heterogeneity and allowing studies of matched tumor and healthy tissues from individual patients [134, 139]. These features make PDOs preferable in vitro tools for disease modeling, drug development, and personalized medicine approaches [158]. To date, several CRC PDO biobanks have been established to pursue these purposes [63, 99, 134, 159]. Seminal studies have shown that CRC PDOs preserve many of the histopathologic, genetic, transcriptomic, and proteomic profiles of the native tumors [63, 99, 134, 147, 159, 160] and recapitulate patient responses in the clinic [63, 148]. Bolhaqeiro et al. exploited CRC PDOs to evaluate the dynamic cell phenotypes in human tumors. Chromosomal instability, involved in tumor evolution and response to therapy, was widespread in CRC and subject to regional variation within PDO, contributing to karyotype heterogeneity [149]. In advanced gastrointestinal tumors, Vlachogiannis et al. demonstrated that PDOs accurately predict targeted therapy responses in patients, outperform molecular biomarkers, and, in co-clinical trials, reflect tumor behavior at baseline, response, and disease progression [63]. Similarly, Ooft et al. showed that mCRC PDOs predicted the patient’s sensitivity to chemotherapy [146]. Treatment with 5-fluorouracil (5-FU)-based chemotherapy induced cancer stem cells (CSCs) activation and enrichment via p53-mediated transcriptional activation of WNT3 followed by activation of WNT/β-catenin pathway. The combinatorial treatment of WNT inhibitor and 5-FU in PDOs and PDX revealed a reduction of CSCs and tumor regrowth, making it a potential therapeutic strategy to overcome the current poor outcomes of 5-FU-based treatment [150]. Recent studies have shown PDO as a helpful approach to predict clinical responses to chemoradiation [135, 160] and validate novel therapeutic strategies [150, 151]. A biobank generated from 80 rectal cancer organoids (RCOs) derived from patients in phase III clinical trial and treated with neoadjuvant chemoradiation was subjected to irradiation combined with 5-FU and CPT-11 treatment, and finally matched with the patient clinical outcomes. The authors found that 16 patients with RCOs sensitive to irradiation obtained a good response with neoadjuvant chemoradiation (NACR). Among 64 patients with RCOs resistant to irradiation, 42 patients had a poor response to NACR and 22 a good response. A good clinical response to chemoradiation was observed in patients whose RCOs were sensitive to at least one treatment. The patients’ clinical outcome highly correlated with RCOs response to therapy with 84.43% accuracy, 78.01, and 91.97% specificity [135]. These promising results encourage the use of CRC PDOs as PDX substitutes for pre-clinical studies aiming at developing patient-specific treatment regimens, with timelines compatible with therapeutic decision-making [152, 161]. Nevertheless, the lack of tumor microenvironment in organoid cultures affects the assessment of therapies targeting the tumor stroma, e.g., the antiangiogenic regorafenib [63]. Therefore, PDXs remain the gold standard for the final validation of drug sensitivity [148, 152]. Increasing numbers of biobanked organoids may help standardize experimental pipelines to predict clinical responses to treatment. Methods for automated culture and HTS have been optimized for kidney organoids [162] and ovarian cancer PDOs [163]. Machine learning approaches from drug responses in CRC organoids have been developed to predict chemotherapy sensitivity in patients accurately [164] and optimize high-throughput image-based drug screening platforms [165]. Since single-cell clonal expansions and genome engineering approaches can be easily performed, both healthy and tumor PDOs have been exploited to study intra-tumor heterogeneity, as well as to interrogate the mutational processes underlying tumor initiation and progression [133, 158].

Clonal organoid cultures can be used as proxies that reflect the genetic make-up of the single cells from which they originate, circumventing the technical limitations of single-cell-based sequencing [139, 166]. Roerink et al. performed an integrated genetic, epigenetic, transcriptomic, and functional analysis in CRC clonal organoids [166], identifying distinct clonal organoids derived from the same tumor region with differential drug responses. To model cancer progression, two independent studies exploited CRISPR/Cas9 gene editing to sequentially introduce mutations in tumor suppressor genes (*APC*, *TP53*, and *SMAD4*) and oncogenes (*KRAS* and *PI3KCA*) in human normal colon organoids. Engineered organoids grew independently of niche factors in vitro and were tumorigenic in vivo [153, 167] but developed spontaneous metastases only after orthotopic transplantation [154, 155]. Fumagalli et al. demonstrated that the majority of CRC metastases were seeded by Leucine Rich Repeat Containing G Protein-Coupled Receptor 5 (Lgr5; a marker of functional stem cells) negative cells that switched into Lgr5 positive cells after metastatic colonization [156]. These data collectively demonstrated that the native microenvironment plays a crucial role in the metastatic process, and niche-independent stem cell plasticity is required for metastatic seeding [154, 156]. PDO model applications, outcome, and limits are summarized in Table 1.

## Patient-derived model: limits and perspectives

Although the PDX model represents an innovative and effective pre-clinical tool as a predictive model of carcinogenesis, individualized cancer therapy, and drug development, large-scale screening of PDXs is limited by the high costs, the long periods (at least 3 months), and the high number of animals required for PDX development. Moreover, the progressive loss of human stromal and immune cells over time in PDXs could significantly impact therapy response. Several studies demonstrated the loss of human stromal cells is replaced by murine counterparts while maintaining the original tumor architecture [52, 60]. At early PDX passages (P0-P4), a fraction of human stromal transcripts is replaced by orthologous mouse stromal cells proportionally to tumor mass. Interestingly, murine stromal cells acquiring a human-like metabolic profile support tumor development and proliferation [52, 60]. Nevertheless, the depletion of stroma-derived signals is likely the major source of transcriptional variation between surgical specimens and PDXs [157]. Therefore, the lack of tumor microenvironment wanes the enthusiasm to use the Patient-derived models because the interaction of the tumor with stromal and immune cells impacts tumor proliferation, apoptosis, and differentiation and on all the mechanisms related to tumor progression. These limitations particularly impact the PDO model, whose main limitation, besides stroma and immune cells, remains the absence of blood vessels [133, 168, 169]. The Patient-derived tumor models allow to reflect the original tumor more accurately and, consequently, to progress on the knowledge of mechanisms related to tumor progression and on new therapies.

### Tumor microenvironment: new patient-derived models upgrade

The context in which tumor develops and the dynamic interaction between the cancer cells and the microenvironment composed by extracellular matrix components, tumor stroma (fibroblasts, myofibroblasts, adipocytes, endothelial cells, pericytes), and immune cells (innate and adaptive immune cells) are fundamental elements for tumor growth, progression, and survival as well as to predict tumor response to therapy.

To study the tumor ecosystem involvement in the chemotherapy and antitumor drug outcomes, Majumder et al. developed a CANScript technology based on thin sections of tumor-derived from patients cultured on tissue well plate coated with different grade-matched tumor matrix support, allowing the conservation of the tumor heterogeneity and architecture [170]. As the tumor-stromal matrix proteins (TMPs) composition was distinct across tumor types and grades, tumor- and grade-specific TMPs were generated. Several pieces of evidence confirmed the vital role at the phenotypic level of TMPs in tumor survival and proliferation. In combination with the matrix support to preserve the phenotype and the molecular features of the naïve tumor, tumors were cultured with the autologous patient serum (AS) containing growth factors and endogenous ligands. Moreover, a positive correlation with cetuximab response was found in the PDX model and the matched CANScript platform. CANScript platform preserved the naïve tumor characteristics such as proliferation, cell viability, EMT, immune cells and cytokines specific marker, and a high degree of the conserved transcriptomic profile of the native tumor. Of note, CANScript technology provides a predictive tool for potential therapeutic responders across different tumor types thanks to the associated algorithm that achieved 100% sensitivity and 91,67% specificity in predicting the clinical responses [170]. The major components of the tumor microenvironment are the cancer-associated fibroblasts (CAFs) that play a crucial role in carcinogenesis and tumoral progression [171, 172]. CAFs are involved in producing cytokines and chemokines that promote the crosstalk with immune cells in matrix deposition and remodeling that lead to tumor stiffness and secretion of soluble factors such as exosomes and growth factors. To reproduce tumor microenvironment in PDO model, maintaining the molecular characterization of the native tumor, Luo et al. encapsulated CRC PDO in a 3D hyaluronan (HA)-gelatin hydrogel that mimics the composition of CRC extracellular matrix (rich in HA and collagen type I) [173]. To recapitulate the CRC-CAF crosstalk, patient-derived CAFs were seeded on the top of hydrogel-based CRC PDO. The generation of PDO-CAFs co-culture enhanced the PDO growth and led to the recovery of biological pathways present in the patient tumor lost in PDO culture alone. Moreover, standard-of-care drugs such as capecitabine, 5-FU, oxaliplatin and irinotecan were tested on CRC PDO-CAFs, showing an enhanced PDO resistance to the drugs [173]. A recent study has further revealed the downregulation in PDOs of genes related to extracellular matrix organization, blood vessel development and lymphocyte activation, reflecting the absence of tumor microenvironment cellular components compared to native tumors [174]. However, PDOs maintained the expression profile of intestinal epithelial-stemness-related genes present in the original tumor. Naruse et al. generating a co-culture based on a PDO chamber system with CAFs, observed that PDO cell viability increased in co-culture conditions compared to the single cultured organoids, providing evidence that the CAFs played an essential role in tumor cell proliferation and anti-apoptotic effects. The transcriptomic analysis revealed 117 genes upregulated in PDO-CAFs co-culture with expression levels comparable to the native tumor, but not in PDOs alone. Among the upregulated genes were found the *REG* (Regenerating gene) family and *DUOX* (Dual oxidase gene) family, known to be involved in cell proliferation, anti-apoptotic functions, EMT process, and drug resistance. Notably, a change in the expression level of REG1 and DUOX2 was observed using different patient CAFs derived from each CRC case [174]. These studies underline how the co-culture system of PDO-CAFs provides a vital tool to mimic the tumor microenvironment. Therefore, the use of this approach may represent an advantage for the study of tumorigenesis. Accordingly, in a recent study, Roulis et al. have investigated the mechanisms underlying colorectal tumorigenesis in co-cultures of mouse intestinal organoids and fibroblasts, identifying a rare fibroblast subpopulation controlling tumor-initiating stem cells through a druggable paracrine pathway [175].

Of course, the lack of an immune-competent environment represents another significant limitation blinding the human immune system’s role and tumor microenvironment-immune cells interaction in tumor progression and metastasis. The generation of “humanized mice” [48, 84, 176] by engraftment with human hematopoietic stem cells (CD34^+^ cells) may represent an attempt to overcome this obstacle. Newborn (1–3 days old) or young (3–6 weeks old) immunodeficient mice can be reconstituted between 4 and 24 h after irradiation with 3 × 10^4^-1 × 10^5^ human CD34^+^ hematopoietic stem cells derived from bone marrow (BM) or umbilical cordon blood (UCB) [177, 178]. Greater than 25% human CD45^+^ cells in the peripheral blood are considered a satisfactory humanization. This occurs in 4–6 weeks [179] or up to 12 weeks [130, 180] post engraftment, depending on whether fresh isolated or cultured CD34^+^ cells are used for the humanization procedure. After an established humanization, PDXs are implanted to generate Human-PDX (HuPDX) models. Capasso et al. exploited the Hu-CRC PDX model to study the immune system’s role in cancer and the efficacy of different immunotherapies [130]. The authors observed that anti-PD-1 therapy (nivolumab), agents that target immune regulatory checkpoints, inhibited tumor growth in MSI-H CRC, correlating with an increase of human T cells, in particular CD8 T cells and INF-γ-producing CD8^+^ tumor-infiltrating leukocytes (TILs). On the contrary, the immunotherapy failed in non-humanized MSI-H CRC PDX mice. Like what happens in human patients treated with anti-PD-1 therapy, the authors observed in MSS CRC Hu-PDX mice that nivolumab led to an initial inhibition of tumor growth, followed by a rapid tumor progression. An outstanding question related to the humanized model remains the human leukocyte antigen (HLA)-mismatched between tumor and donor immune cells. However, some evidence reports no correlation between HLA and the engraftment of PDX [130, 179]. Ideally, the best condition to generate the Hu-PDX model would be to use the same patient’s immune system from whom the tumor is collected to develop the PDX model, maintaining the tumor microenvironment as similar as possible to the original tumor. However, although humanized mice provide new and incredible avenues, further studies are necessary to set human adult CD34 engraftment and immune reconstitution. CRC organoid co-culture systems with autologous tumor-reactive T cells [181] or chimeric antigen receptor (CAR)-engineered lymphocytes [182] as well as air-liquid interface (ALI) methods [183], have been implemented to address the limits of Hu-PDX. Neal et al. to preserve the stromal cells and tumor-specific TILs in the PDO developed ALI PDO methods [183]. In the ALI system, minced tumor tissues embedded in type I collagen gel was grown on the top of a transwell insert containing a bottom permeable and membranous layer composed of collagen matrix and concentrate sterile culture medium. The transwell was placed into a larger cell culture dish containing the appropriate medium. In this system, PDOs preserved fibroblast stroma that progressively decreased with the passages and contained CD3^+^ T cells in proximity to tumor epithelium, CD14^+^, CD68^+^ NK, and B cells. The PDO TILs, although progressively decreased over 60 days, represented the immune cell population and T cell receptor repertoire of the original tumor. The PDO TILs activities were evaluated by treatment with anti-PD-1 or anti-PD-L1 in murine tumor organoids that showed a strong increase of CD8 TILs and T cell activation markers such as interferon-gamma (IFNγ), perforin-1 (PRF1), and granzyme B recapitulating the PD-1/PD-L1 immune checkpoint [183]. ALI PDO system may allow the development of personalized cancer therapy thanks to incorporating stromal and immune cells. PDOs derived from chemotherapy-resistant metastatic CRCs were co-culture with allogeneic CD8^+^ T cells by Gonzalez-Exposito et al. to evaluate the efficacy of the T cell bi-specific antibody cibisatamab (CEA-TCB). CEA-TCB binding carcinoembryonic antigen (CEA) on cancer cells and CD3 on T cells mediate cancer cell recognition and T cells’ killing. High CEA expression in PDOs was determinant for the cibisatamab efficacy, while a low CEA expression correlated with WNT/β-catenin pathway activity and resistance to immunotherapy [184]. Despite the tremendous advances, Patient-derived models still present important limitations as the lack of autologous CAFs for co-culture with the matched PDOs whose composition has been demonstrated important considering the inter-variability between tumors and patients and the lack of autologous immune system for the generation of humanized mice and co-culture with PDOs. Moreover, it is relevant to consider in PDX mouse model the lack of a comparable human microbiota, key player in tumor development and progression, due to the housing condition of mice including the standard diet on which mice are fed [185, 186]. Although there is a reasonable agreement that PDX could maintain the genomic fingerprints of their matched donor samples, the debate is still open. Indeed, some authors argue that the clonal drifting of these models may occur. We believe that driving mutations remain faithfully represented in the PDX. In the last few years innovative methodologies have been developed and optimized more and more, in order to overcome the Patient-derived model limitations, making these models increasingly faithful and able to recapitulate the biological mechanism involved in tumor development and progression. However, few studies have comprehensively investigated their genome (WES or WGS) and systematically interrogated the emergency of (even minor) defects over extensive passages. Moreover, the epigenome of these models remains mostly not explored. While large-scale chemical and genetic perturbation approaches (e.g. CRISPR/Cas9 screens) in PDOs will be further implemented, it is reasonable to hypothesize that organoid-based personalized medicine may support clinical decision making in real time with patient care (e.g. co-clinical trials). In parallel, PDOs may be also exploited to model patients’ responses and test mechanistic hypotheses. So far, few preliminary studies in metastatic pancreatic cancer suggested the possibility to generate organoids from circulating tumor cells [187–189]. If confirmed for this and other tumor types, including CRC, this approach may be particularly useful to reduce repeated tissue biopsies during longitudinal clinical studies.

Over the next decade, strategies aiming to integrate patient-derived models, single-cell omics, advanced imaging approaches, and artificial intelligence will be implemented to improve early cancer detection, selection of the most effective therapeutic strategies, and prediction of acquired resistance [190].

## Conclusion

CRC is a multistep process determined by the combination of multiple genetic and epigenetic aberrations and environmental risk factors. The research for new therapeutic strategies remains an ongoing challenge for CRC due to its considerable intra- and inter-tumoral phenotypic heterogeneity characterized by different molecular features and responses to therapy, which are not reflected in preclinical models such as tumor cell lines and GEMMs. The biological inadequacy of preclinical models leads to the failure of clinical trials. PDX and PDO are promising and innovative preclinical tools to study the onset, progression, and metastasis of CRC and investigate the primary and acquired mechanisms of resistance to therapy, understanding the cellular clonal evolution during tumor growth. Moreover, these models allow the generation of drug screening platforms to develop and test new therapeutic drugs and predict the clinical outcomes of the therapy, identifying prognostic and predictive biomarkers (personalized medicine). In this scenario, the peculiarities of these models can be exploited for interchangeable applications: while PDOs are more amenable than PDXs for high-throughput screens, less time-consuming and more cost-effective, in vivo models remain the gold standard for final validation of drug efficacy; on the other hand, biologically relevant findings in PDXs may be further mechanistically investigated in vitro using PDX-derived organoids. The possibility to effectively establish PDOs from PDXs and vice versa allows generating matched PDO-PDX collections easily. This, in turn, may help in obtaining a full *armamentarium* of patient-derived experimental models even when a reduced amount of tumor material is available. Despite their growing relevance in CRC study and therapy, Patient-derived models have some relevant limitations related to the lack of human immune and stromal cells that, by interacting with the tumor cells, contribute to the tumoral progression. To overcome these limits, new methodological strategies have been developed. To reproduce the human tumor microenvironment-immune cells interaction in PDX, humanized PDX mouse model has been generated, while to address the absence of stroma and immune cells in PDO, air-liquid interface methods and co-culture systems have been implemented. However, with their continuous optimizations and implementations, the Patient-derived models represent the most promising CRC preclinical model to dissect the multifactorial etiology and progression of the tumor and develop personalized therapy based on the patients’ features.

## Data Availability

Not applicable.

## Abbreviations

CRC: Colorectal cancer
FAP: Familial adenomatous polyposis
MSS: Microsatellite Stable
MSI: Microsatellite Instability
GEMMs: Genetically engineered mouse models
APC: Adenomatous polyposis coli
Min: Multiple intestinal neoplasia
AOM: Azoxymethane
DSS: Dextran sulfate sodium
PDX: Patient-derived xenograft
CMS: Consensus molecular subtype
PDO: Patient-derived organoid
NGS: Next-generation sequencing
PTEN: Phosphatase and tensin homolog
PIK3CA: Phosphatidylinositol-4,5-bisphosphate 3-kinase catalytic subunit alpha
5-FU: 5-fluorouracil
RT: Radiotherapy
s.c.: Subcutaneous implantation
NOD/SCID: Nonobese diabetic/severe combined immunodeficiency
NSG: NOD/SCID/IL2γ-receptor null
Sirpa: Signal regulatory protein alpha
ERBB2: Erb-B2 receptor Tyrosine kinase 2
EGFR: Epidermal growth factor receptor
HER2: Human epidermal growth factor receptor 2
FGFR1: Fibroblast growth factor receptor 1
MAP2K1: Mitogen-activated protein kinase 1
IGF2: Insulin like growth factor 2
EML4: Echinoderm microtubule-associated protein-like 4
ALK: Anaplastic lymphoma kinase
GSK3α: Glycogen Synthase Kinase 3α
shRNA: Short hairpin RNA
HTS: High-throughput screening
LGR5: Leucine Rich Repeat Containing G Protein-Coupled Receptor 5
BM: Bone marrow
UCB: Umbilical cordon blood
Hu-PDX: Human-PDX
PD-1: Programmed Cell Death 1
INF: Interferon
TILs: Tumor-infiltrating leukocytes
CAR: Chimeric antigen receptor
CEA: Carcinoembryonic antigen
CEA-TCB: T cell bi-specific antibody cibisatamab
zPDX: Zebrafish PDX
CSCs: Cancer stem cells
RCOs: Rectal cancer organoids
NACR: Neoadjuvant chemoradiation
MATE1: Multidrug and toxic compound extrusion 1
CAR-T: Chimeric Antigen Receptor T
TMPs: Tumor-stromal matrix proteins
AS: Autologous patient serum
CAFs: Cancer-associated fibroblasts
*REG*: Regenerating gene
*DUOX*: Dual oxidase gene
HLA: Human leukocyte antigen
